# EPCR deficiency ameliorates inflammatory arthritis in mice by suppressing the activation and migration of T cells and dendritic cells

**DOI:** 10.1093/rheumatology/kead230

**Published:** 2023-05-25

**Authors:** Meilang Xue, Haiyan Lin, Hai Po Helena Liang, Lara Bereza-Malcolm, Tom Lynch, Premarani Sinnathurai, Hartmut Weiler, Christopher Jackson, Lyn March

**Affiliations:** Sutton Arthritis Research Laboratory, Sydney Musculoskeletal Health, Kolling Institute, Faculty of Medicine and Health, The University of Sydney and the Northern Sydney Local Health District, Sydney, NSW, Australia; Australian Arthritis and Autoimmune Biobank Collaborative (A3BC), Sydney Musculoskeletal Health, Kolling Institute, Faculty of Medicine and Health, The University of Sydney and the Northern Sydney Local Health District, Sydney, NSW, Australia; Sutton Arthritis Research Laboratory, Sydney Musculoskeletal Health, Kolling Institute, Faculty of Medicine and Health, The University of Sydney and the Northern Sydney Local Health District, Sydney, NSW, Australia; Australian Arthritis and Autoimmune Biobank Collaborative (A3BC), Sydney Musculoskeletal Health, Kolling Institute, Faculty of Medicine and Health, The University of Sydney and the Northern Sydney Local Health District, Sydney, NSW, Australia; Sutton Arthritis Research Laboratory, Sydney Musculoskeletal Health, Kolling Institute, Faculty of Medicine and Health, The University of Sydney and the Northern Sydney Local Health District, Sydney, NSW, Australia; Sutton Arthritis Research Laboratory, Sydney Musculoskeletal Health, Kolling Institute, Faculty of Medicine and Health, The University of Sydney and the Northern Sydney Local Health District, Sydney, NSW, Australia; Australian Arthritis and Autoimmune Biobank Collaborative (A3BC), Sydney Musculoskeletal Health, Kolling Institute, Faculty of Medicine and Health, The University of Sydney and the Northern Sydney Local Health District, Sydney, NSW, Australia; Australian Arthritis and Autoimmune Biobank Collaborative (A3BC), Sydney Musculoskeletal Health, Kolling Institute, Faculty of Medicine and Health, The University of Sydney and the Northern Sydney Local Health District, Sydney, NSW, Australia; Australian Arthritis and Autoimmune Biobank Collaborative (A3BC), Sydney Musculoskeletal Health, Kolling Institute, Faculty of Medicine and Health, The University of Sydney and the Northern Sydney Local Health District, Sydney, NSW, Australia; Versiti Blood Research Institute, Versiti, Milwaukee, WI, USA; Department of Physiology, Medical College of Wisconsin, Milwaukee, WI, USA; Sutton Arthritis Research Laboratory, Sydney Musculoskeletal Health, Kolling Institute, Faculty of Medicine and Health, The University of Sydney and the Northern Sydney Local Health District, Sydney, NSW, Australia; Australian Arthritis and Autoimmune Biobank Collaborative (A3BC), Sydney Musculoskeletal Health, Kolling Institute, Faculty of Medicine and Health, The University of Sydney and the Northern Sydney Local Health District, Sydney, NSW, Australia

**Keywords:** endothelial protein C receptor, RA, CIA, Th cells, dendritic cells, cytokines

## Abstract

**Objectives:**

Endothelial protein C receptor (EPCR) is highly expressed in synovial tissues of patients with RA, but the function of this receptor remains unknown in RA. This study investigated the effect of EPCR on the onset and development of inflammatory arthritis and its underlying mechanisms.

**Methods:**

CIA was induced in EPCR gene knockout (KO) and matched wild-type (WT) mice. The onset and development of arthritis was monitored clinically and histologically. T cells, dendritic cells (DCs), EPCR and cytokines from EPCR KO and WT mice, RA patients and healthy controls (HCs) were detected by flow cytometry and ELISA.

**Results:**

EPCR KO mice displayed >40% lower arthritis incidence and 50% less disease severity than WT mice. EPCR KO mice also had significantly fewer Th1/Th17 cells in synovial tissues with more DCs in circulation. Lymph nodes and synovial CD4 T cells from EPCR KO mice expressed fewer chemokine receptors CXCR3, CXCR5 and CCR6 than WT mice. *In vitro*, EPCR KO spleen cells contained fewer Th1 and more Th2 and Th17 cells than WT and, in concordance, blocking EPCR in WT cells stimulated Th2 and Th17 cells. DCs generated from EPCR KO bone marrow were less mature and produced less MMP-9. Circulating T cells from RA patients expressed higher levels of EPCR than HC cells; blocking EPCR stimulated Th2 and Treg cells *in vitro*.

**Conclusion:**

Deficiency of EPCR ameliorates arthritis in CIA via inhibition of the activation and migration of pathogenic Th cells and DCs. Targeting EPCR may constitute a novel strategy for future RA treatment.

Rheumatology key messages

- EPCR deficiency reduces the incidence as well as severity in the mouse collagen-induced arthritis model.
- EPCR deficiency reduces the activation and migration of inflammatory Th cells and dendritic cells.
- EPCR was overexpressed by T cells from patients with rheumatoid arthritis.

## Introduction

RA is an autoimmune disease characterized by progressive joint inflammation and destruction. This inflammation/damage is mediated by a highly coordinated innate and adaptive immune system, including dendritic cells (DCs), B cells, T cells and their autoantibodies and cytokines [1, 2]. In particular, CD4 T cells are able to differentiate into functionally distinct cell types, such as Th1, Th2, Th17 and Treg cells. The balance of these T cell subsets is critical for RA onset and development [3, 4].

Endothelial protein C receptor (EPCR; CD201) is a transmembrane protein expressed by many cell types, including most immune cells, endothelial and epithelial cells, fibroblasts and chondrocytes. It is a key receptor for natural anticoagulant-activated protein C (aPC), facilitating aPC’s anti-clotting, anti-inflammatory and barrier protective functions [5]. In addition to aPC, EPCR can also interact with other ligands, including T cell receptor [6], aPLs [7] and secretory phospholipase A2 (sPLA_2_) [8], exhibiting ligand-dependent function. Furthermore, EPCR is homologous to MHC class I/CD1 family proteins [9], a group of antigen-presenting molecules for immune surveillance by T cells, with the potential to regulate both innate and adaptive immunity. EPCR can inhibit Th17 cells and T cell–specific deficiency of EPCR results in the exacerbation of experimental autoimmune encephalomyelitis (EAE) in mice [10]. However, in patients with psoriasis, EPCR on circulating T cells positively correlates with disease severity and anti-TNF treatment reduces EPCR expression and disease severity [11]. Higher levels of EPCR can predict poor outcomes of colorectal and lung cancers [12, 13], severe lung infection and inflammation [14] and poor treatment response of patients with lupus nephritis [15]. These data suggest that EPCR can regulate T cell function and may be a potential therapeutic target for inflammatory/autoimmune diseases.

In RA, EPCR is strongly expressed in the pannus, the hypertrophied tissue that contributes to joint destruction in RA [8, 16]. *In vitro*, binding of EPCR to sPLA_2_ impairs the endothelial barrier [17] and promotes the growth/invasion of RA synovial fibroblasts, the major cell type within the pannus [8]. Conversely, EPCR protects against mouse inflammatory arthritis when it acts as a receptor for aPC [16]. Plasma levels of EPCR, a cleaved form of cell membrane EPCR, are higher in patients with RA [16] when compared with healthy controls (HCs). Despite these emerging data, whether EPCR directly contributes to RA has never been investigated. Using EPCR knock-out (EPCR KO) mice, this study demonstrated that EPCR deficiency prevents arthritis onset and mitigates disease severity in CIA, a gold standard model of human RA. These data suggest that overexpression of EPCR likely contributes to RA pathogenesis.

## Materials and methods

### Mice

Male EPCR KO (C57BL/6J Meox2Cre-EPCRloxP) mice and matched wild-type (WT) mice were obtained from the Kearns Facility, Kolling Institute, University of Sydney. EPCR KO mice appeared healthy and no haemorrhage or visible evidence of spontaneous thrombosis was observed [18]. Genotypes of mice were confirmed by PCR using genomic DNA from the tip of the mouse tail.

### CIA model

Male EPCR KO and WT mice (10–12 weeks old) were immunized with chicken type II collagen (CII) in complete Freund’s adjuvant at day 0 and day 21 as described previously [16]. After the second immunization, mice were examined for clinical signs of arthritis in the wrist and ankle joints and scored visually using a previously described severity scale [16]. Scoring was conducted under blinded conditions.

Breeding and use of animals and all procedures were approved by the Royal North Shore Hospital Animal Ethics Committee and Institutional Biosafety Committee.

### Patients and patient samples

Six RA patients who fulfilled the 2010 ACR/EULAR RA classification criteria and matched HCs were recruited from Royal North Shore Hospital. HCs had no history of any autoimmune disorders. Peripheral blood was taken from these patients and peripheral blood mononuclear cells (PBMCs) were isolated. PBMCs were used for detecting EPCR expression or treated with TNF inhibitors [adalimumab (ADA) and etanercept (ETA)], EPCR blocking antibody RCR252 or non-blocking antibody RCR92 for 24 h. The use of human tissues was approved by the Northern Sydney Local Health District Human Research Ethics Committee. All patients gave their written informed consent.

### Histological analysis and immunohistochemical staining

Mice were euthanized at designated time points and blood, inguinal lymph nodes, spleens and knee joints were harvested. Knee joints were either used for synovial cell isolation or fixed and embedded in paraffin for histological/immunohistochemical examination. Joint sections were stained with haematoxylin and eosin or toluidine blue. Cartilage erosions and synovial inflammation were evaluated as described previously [19].

### Immunohistochemical staining

Mouse joint sections were de-paraffinized and subjected to immunostaining using rabbit anti-mouse PC/aPC (R&D Systems, Minneapolis, MN, USA) after heat retrieval. Tissue sections were then incubated with appropriate horseradish peroxidase–conjugated secondary antibodies, followed by Liquid DAB+ Substrate Chromogen System staining (Agilent DAKO, Santa Clara, CA, USA). Finally, tissue sections were counterstained, mounted and photographed under a microscope. Anti-rabbit isotype IgG was used as a negative control.

### Flow cytometric detection of Th cells, DCs and other immune cells

Mouse synovial tissues were diced and dissociated by Liberase (Merck, Darmstadt, Germany) digestion. Single cell suspensions of synovial tissue, blood, lymph nodes and spleen were stained with mouse antibody panels to identify immune cell populations as described previously [16]; CD3-AF700, CD4-BV711, T-bet-BV786, GATA3-PECY7, Ror-γ-BV421, FOX3-PE, CD25-APC and/or CD201-PE (EPCR) to identify Th1/Th2/Th17/Treg cells and EPCR expression on these cells; CD11C-PE-CF594, CD317-BV421, CD45R/B220-APC-H7, CD11b-BB515, I-Ab-BV711, CD80-BV605, CD86-PE-Cy7 and CD40-APC to identify DCs and DC subsets [total DC-CD11c^+^, myeloid (m)DC-CD11c^+^CD11b^+^B220^+^, plasmacytoid (p)DC-CD11c^+^CD11b-B220^+^PDCA-1^+^, conventional (c)DC-CD11b+CD11c^+^, DC maturation (CD40^+^, CD80^+^, CD86^+^, IAb (I-Ab MHC class II alloantigen)]; and CD4-BV711, CXCR3 (CD183-APC), CXCR5 (CD185-PE-Cy7), CCR6 (CD196-BV421) and CCR7 (CD197-BV650) to detect chemokine receptor expression on T cells. Human CD3-AlexaFluor 700, CD4-APC-H7 and EPCR-PE were used to detect EPCR expression on CD3 and CD4 T cells; human CD3-Alexa Fluor 700, CD4-APC-H7, CCR4-BV421, CCR6-BV786, CCR10-APC, CD25-BB515, CD127-BV711 and CXCR3-PE-Cy7 to identify Th1 (CD3^+^CD4^+^CD25^−^ CXCR3^+^CCR6^−^), Th2 (CD3^+^CD4^+^CD25^−^CXCR3^−^CCR6^−^CCR4^+^), Th17 (CD3^+^CD4^+^CD25^−^ CXCR3^−^CCR6^+^CCR4^+^CCR10^−^) and Treg cells (CD3^+^CD4^+^CD25^+^CD127^−^). All antibodies were purchased from BD (North Ryde, NSW, Australia). Detection and data analysis were performed using a BD LSRFortessa flow cytometer.

### aPC activity

Synovial tissues obtained from mouse knee joints were homogenized and collected into protein extraction reagent (Pierce, Rockford, IL, USA). After vortexing, the homogenates were centrifuged and the clear supernatants collected. The activity of aPC in plasma and clear supernatants of synovial tissues was measured by the chromogenic substrate Spectrozyme PCa assay (American Diagnostica, Stamford, CT, USA).

### Generation of bone marrow–derived DCs (BMDCs)

BMDCs were induced using a standard protocol as described previously [20]. At day 7, cells were incubated with lipopolysaccharide (LPS, 1 µg/ml) at 37°C for 24 h to induce DC maturation. Cells were harvested for experiments at day 8.

### Mouse spleen cell treatment

Spleen cells were isolated from mice and treated with anti-EPCR blocking antibody (RCR16) (Thermo Fisher Scientific Australia, Scoresby, VIC, Australia) or non-blocking antibody (RCR20), human recombinant aPC and/or LPS for 24 h. Th cell phenotypes were analysed by flow cytometry.

### ELISA

Mouse anti-CII antibodies (IgG1, IgG2a and IgG2b; Thermo Fisher Scientific Australia), mouse cytokines TGF-β1, IL-4, IL-17, IFN-γ and TNF-α and MMP-3 were measured using ELISA kits (R&D Systems) according to the manufacturer’s instructions.

### Gelatin zymography

MMP-2 and MMP-9 protein secretion and activation in cell culture supernatants were measured using gelatine zymography under non-reducing conditions as described previously [21].

### T cell proliferation

Mouse spleen cells were labelled with 1 μm carboxyfluorescein succinimidyl ester (CFSE) and cultured in 96-well plates coated with anti-CD3 and anti-CD28 antibodies (BD). After 5 days, cells were harvested and directly analysed by flow cytometry.

### Statistical analysis

All experimental results are expressed as mean (s.d.). Comparisons were performed using the two-tailed Student’s *t*-test or one- or two-way analysis of variance (ANOVA) as appropriate. Student’s *t*-test was used for data comparing two data sets only. Significance was set at *P* < 0.05. Survival plots (Kaplan–Meier) and logrank analysis was used to compare arthritis incidence in CIA. All statistical calculations were performed using GraphPad Prism 9 (GraphPad Software, San Diego, CA, USA).

## Results

### EPCR KO mice exhibit lower arthritis incidence and less severe disease in CIA

**Figure 1.**
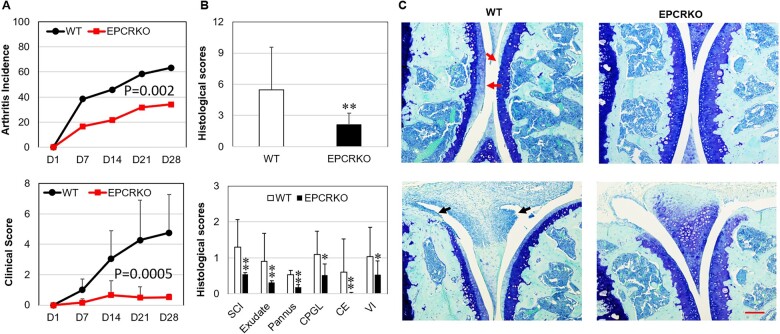
The incidence and severity of CIA in EPCR KO and WT mice. Male EPCR KO and WT mice were immunized with chicken type II collagen on days 0 and 21. Clinical signs of arthritis were examined after the second immunization (arthritis induction), until day 28, when mice were euthanized for histological evaluation. **(A)** Arthritis incidence and average clinical scores in mice (*n* = 25 mice/group). **(B)** Histological scores in mice (*n* = 20 mice/group). SCI: synovitis cell infiltrate; CPGL: cartilage proteoglycan loss; CE: cartilage erosion; VI: vascular invasion from subchondral bone. Data are shown as mean (s.d.). *P < 0.05, **P < 0.01 *vs* WT. **(C)** Representative knee joints from mice with CIA stained with toluidine blue. Arrows in upper panel indicate damaged cartilage and arrows in lower panel indicate synovial invasion. Scale bar: 200 µm

In the CIA model, at day 28 after arthritis induction, ∼ 60% of WT mice developed arthritis (determined by scoring as 0 for no arthritis) whereas only 34% of EPCR KO mice had arthritis (*P* = 0.002; Fig. 1A). Correspondingly, arthritis clinical scores of WT mice were more than nine times higher than that of EPCR KO mice (*P* = 0.0005; Fig. 1A, all mice subjected to two CII immunization were counted). Histological analysis of knee joints confirmed that WT mice displayed significantly higher disease scores for all parameters measured, including exudate, cartilage erosion/loss, pannus formation and vascular invasion, when compared with EPCR KO mice at day 28 after CIA induction (Fig. 1B and C, all mice were counted).

**Figure 2.**
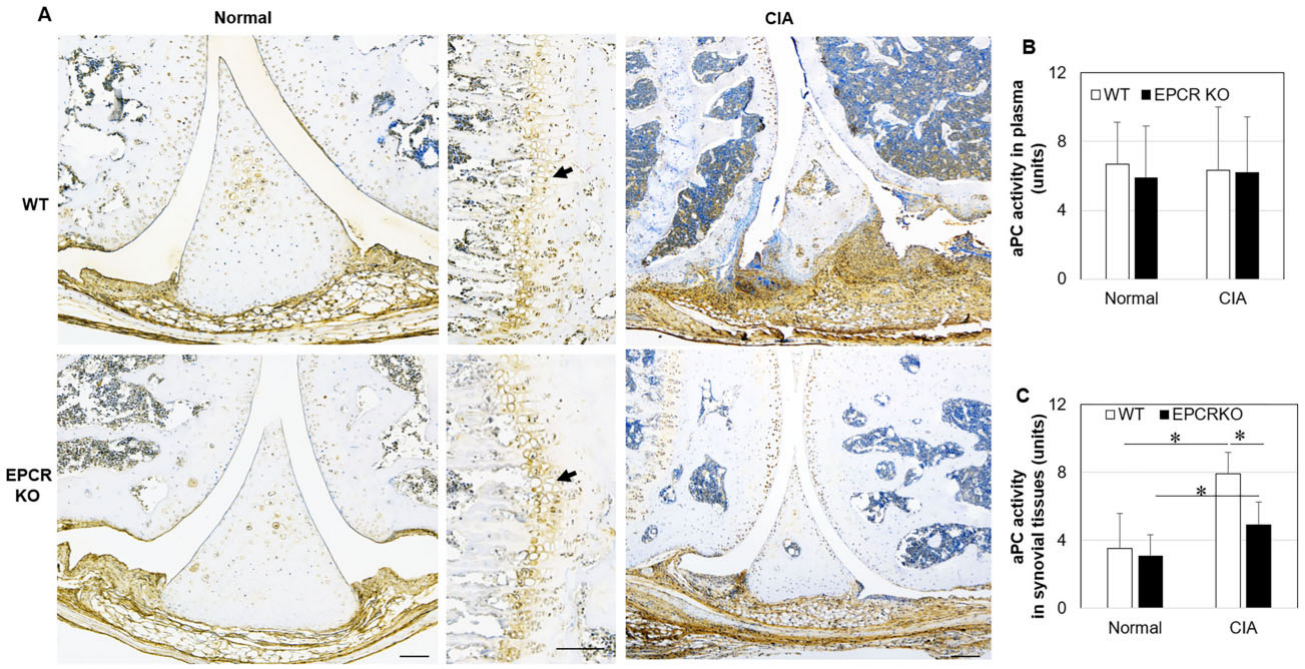
PC/aPC expression/activity in EPCR KO and WT mice. PC/aPC expression/activity in synovial tissues or plasma from normal mice (male, 7 weeks old) or mice with CIA at day 28 after arthritis induction detected by immunostaining (*n* = 10) or aPC activity assay. **(A)** Representative image of PC/aPC expression in synovial tissues, detected by immunostaining. Arrows indicate growth plates in normal mice. Scale bar: 200 µm. **(B, C)** aPC activity in synovial tissues (*n* = 10) or plasma from normal (*n* = 5) or CIA mice (*n* = 20), measured by aPC activity assay. Data are shown as mean (s.d.). aPC activity in synovial tissues is expressed as ng/100 mg tissue and in plasma as ng/ml of recombinant human aPC (units). *P < 0.05 *vs* WT

To examine whether EPCR deficiency affects PC/aPC level, immunostaining and aPC activity assay were performed. In normal joint tissue, PC/aPC was expressed equally by WT and EPCR KO mice, largely in chondrocytes, synovial cells, endothelial cells and the joint growth plate. Overall, PC/aPC expression by chondrocytes and synovial cells was increased in CIA, but less PC/aPC presence was found in the synovial tissue of EPCR KO mice when compared with WT (Fig. 2A). This was confirmed by the aPC activity assay. In CIA, aPC activity was increased in the synovial tissue of all mice, but EPCR KO synovial aPC activity was significantly lower when compared with WT mice (Fig. 2C). aPC activity in plasma did not show a difference between WT and EPCR KO mice in either unchallenged or CIA conditions (Fig. 2B).

### Levels of cytokine, MMP-3, anti-CII antibodies and Th cells in CIA

**Figure 3.**
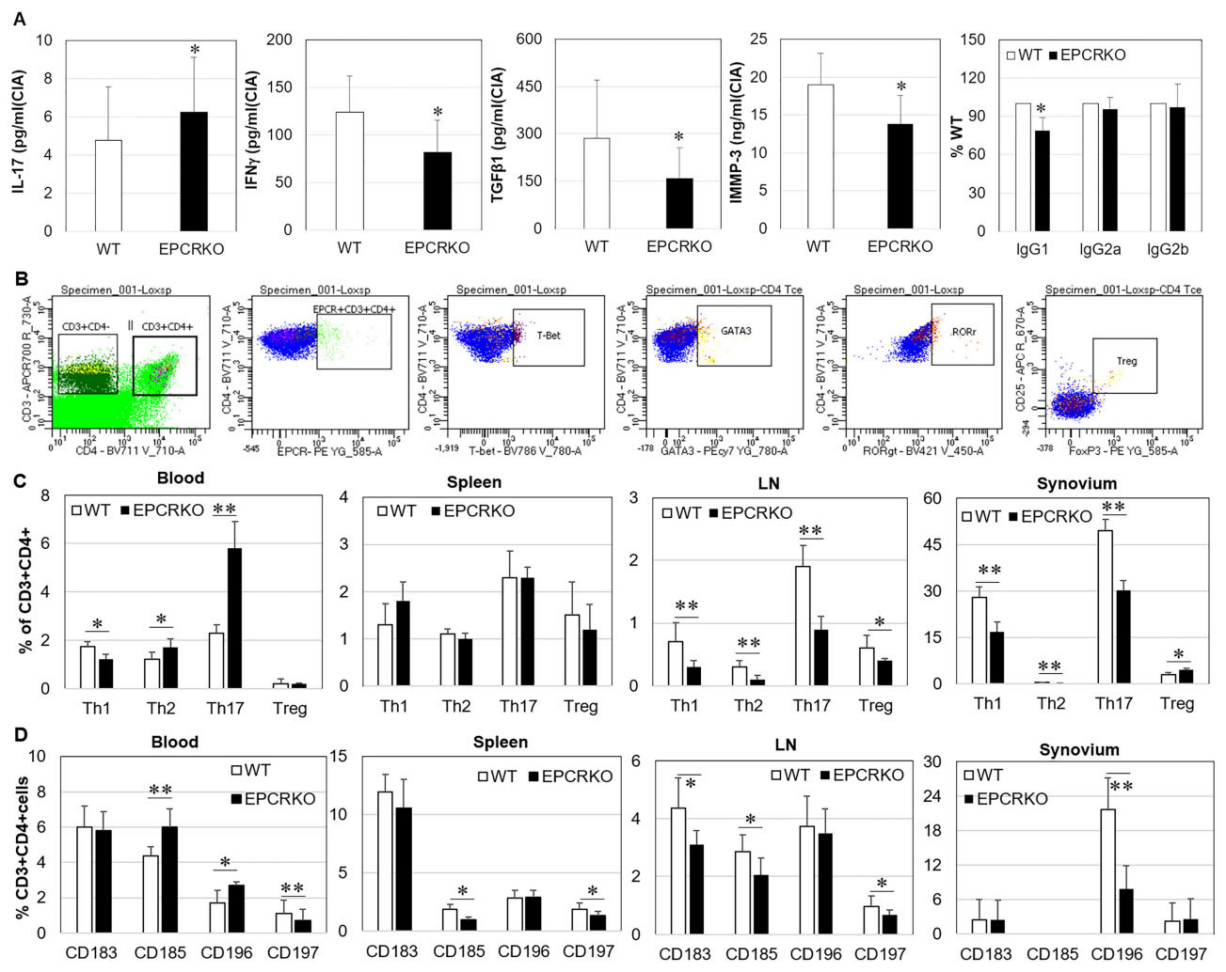
Plasma cytokines and anti-CII antibodies and tissue Th1/2/Th17/Treg cells in mice with CIA. **(A)** IL-17, IFN-γ, MMP-3, TGF-1β and anti-CII antibodies IgG1, IgG2a and IgG2b in plasma from EPCR KO and WT mice with CIA at day 28 after arthritis induction, measured by ELISA (*n* = 20). **(B)** Gate strategies for Th and Treg cell detection by flow cytometry. **(C)** Th1, Th2, Th17 and Treg cells and **(D)** chemokine receptors CXCR3 (CD183), CXCR5 (CD185), CCR6 (CD196) and CCR7 (CD197) on CD3^+^CD4^+^ T cells from blood, spleen, lymph node (LN) and synovium of WT and EPCR KO mice at day 28 after arthritis induction, detected by flow cytometry (*n* = 6). Data are shown as mean (s.d.). *P < 0.05, **P < 0.01 *vs* WT

Inflammatory cytokines associated with RA were detected in mouse plasma at day 28 after CIA induction. In EPCR KO mice, IL-17 was significantly higher but IFN-γ and TGF-β1 lower than in WT animals (Fig. 3A). TNF was not detectable in either WT or EPCR KO mice in both normal and CIA conditions. Plasma anti-CII IgG1 antibody was ∼20% lower, whereas IgG2a and IgG2b were similar in EPCR KO mice when compared with WT (Fig. 3A). Serum MMP-3 levels have been shown to correlate with RA disease activity [22]. Similarly, in CIA, plasma MMP-3 was significantly lower in EPCR KO mice compared with WT mice (Fig. 3A).

Th cells, particularly Th1 and Th17 cells, which produce the signature cytokine IFN-γ and IL-17, respectively, play an important role in the pathogenesis of RA. To examine the effect of deficiency of EPCR, Th and Treg cells within blood, lymph node, spleen and synovial tissue from EPCR KO and WT mice with CIA were analysed by flow cytometry. Fig. 3B shows the gating strategies. Results showed that Th1, Th2 and Th17 cells were significantly higher in blood, lower in lymph node and synovial tissue and not different in spleens from EPCR KO mice when compared with WT mice (Fig. 3C). Treg cells in EPCR KO mice were lower in lymph node, higher in synovial tissue and similar in blood and spleen compared with WT mice (Fig. 3C).

The expression profile of chemokine receptor on CD4^+^ T cells has been shown to correlate with their function. In EPCR KO mice, CD183 (CXCR3) and CD196 (CCR6) on these cells from lymph node or synovial tissue were significantly lower (Fig. 3D); CD185 (CXCR5) was higher in blood and lower in spleen, while it was not detectable in synovium of both WT and EPCR KO mice; and CD197 (CCR7) was not detectable in the blood and lower in lymph node and spleen when compared with WT mice (Fig. 3D).

### The effect of EPCR on spleen T cells *in vitro*

**Figure 4.**
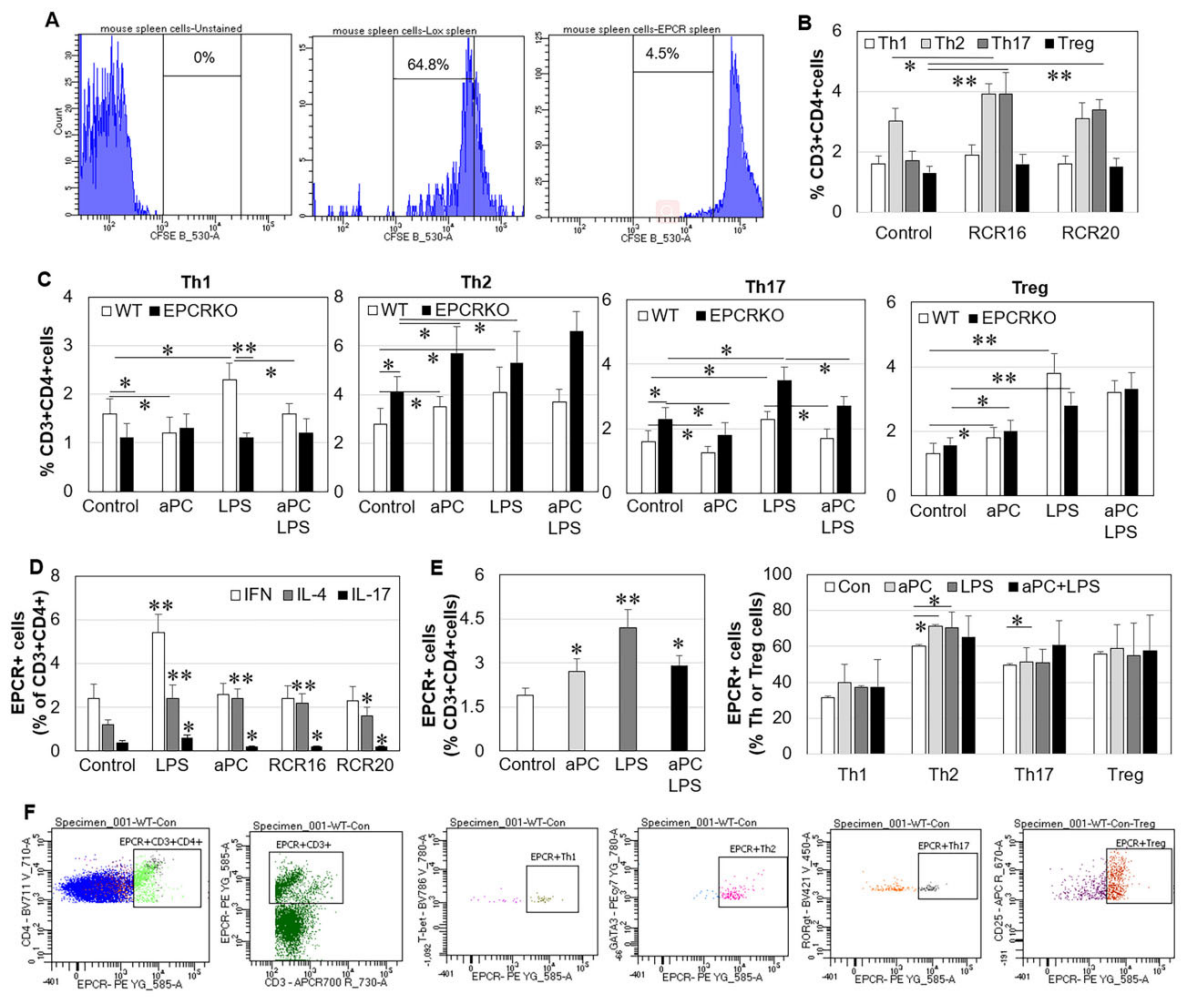
Lymphocyte proliferation and Th phenotypes in WT or EPCR KO spleen cells *in vitro*. Spleen cells isolated from WT or EPCR KO mice were labelled with CFSE or treated with mouse EPCR blocking antibody RCR16 or non-blocking antibody RCR20 (5 µg/ml), aPC (10 µg/ml) and LPS (100 ng/ml) for 24 h. Lymphocyte proliferation or Th phenotypes were detected by flow cytometry. **(A)** Lymphocyte proliferation evaluated by the intense fluorescence of CFSE after a 5 day incubation. **(B, C)** The percentages of Th1, Th2, Th17 and Treg cells in CD3^+^CD4^+^ T cells within spleen cells. **(D)** The percentage of IFN-γ (Th1), IL-4 (Th2) and IL-17 (Th17) producing CD3^+^CD4^+^ T cells. **(E)** EPCR expression by CD3^+^CD4^+^ T cells or Th1/Th2/Th17/Treg cells. **(F)** Gating strategies of EPCR on T cells. Data are shown as mean (s.d.) (*n* = 4). *P < 0.05, **P < 0.01 *vs* controls

To examine the effect of EPCR on T cells *in vitro*, normal WT and EPCR KO mouse spleen cells were isolated and treated. When evaluated by CFSE staining, the proliferation of EPCR KO lymphocytes within spleen cells was significantly lower than in WT after a 5 day incubation (4.5% *vs* 64.8%) (Fig. 4A). This is consistent with the evidence that EPCR is a marker for mouse haematopoietic stem cells [23]. After 24 h incubation, WT spleen cells contained higher levels of Th1 and lower levels of Th2 and Th17 cells than EPCR KO cells (Fig. 4C). In WT cells, RCR16, an EPCR blocking antibody, increased Th2 cells by 30% and Th17 cells by 130%, while having no significant effect on Th1 and Treg cells (Fig. 4B). Surprisingly, the non-blocking antibody RCR20 also showed a similar effect to Th17 cells (Fig. 4B), although to a lesser extent, indicating that RCR20 also exerts a partial blocking effect. In WT cells, aPC decreased Th1 and Th17, while it increased Th2 cells. In EPCR KO cells, aPC increased Th2 cells but had no significant effect on Th1 cells (Fig.4C), suggesting that aPC requires EPCR to suppress Th1. LPS increased Th1, Th2, Th17 and Treg cells in WT cells, but only stimulated Th2, Th17 and Tregs in EPCR KO cells (Fig. 4C). aPC reduced LPS-stimulated Th1 in WT cells and Th17 in both WT and EPCR KO cells. In agreement with these data, LPS increased IFN-, IL-4- and IL-17-producing CD4^+^ cells, while aPC increased IL-4- but decreased IL-17-producing CD4^+^ cells (Fig. 4D). RCR16 showed a similar effect to aPC in WT cells. These data indicate that suppressing EPCR can inhibit Th1 but stimulate Th2 and Th17 cells.

EPCR expression on Th cells was also detected by flow cytometry using anti-EPCR antibody (Fig. 4F shows the gating strategies). In control conditions, ∼2% of CD3^+^CD4^+^ cells within WT spleen cells expressed EPCR (Fig. 4E), while EPCR on CD3^+^CD4^+^ cells within EPCR KO spleen cells was undetectable (data not shown). LPS and aPC significantly stimulated EPCR expression on CD3^+^CD4^+^ cells within WT spleen cells (Fig. 4E). EPCR was expressed by ∼40% of Th1 cells, ∼60% of Th2 cells and ∼50% of Th17 and Treg cells; aPC significantly increased EPCR-expressing Th2 cells, while LPS increased EPCR-expressing Th2 and Th17 cells (Fig. 4E).

### The effect of EPCR on DCs

**Figure 5.**
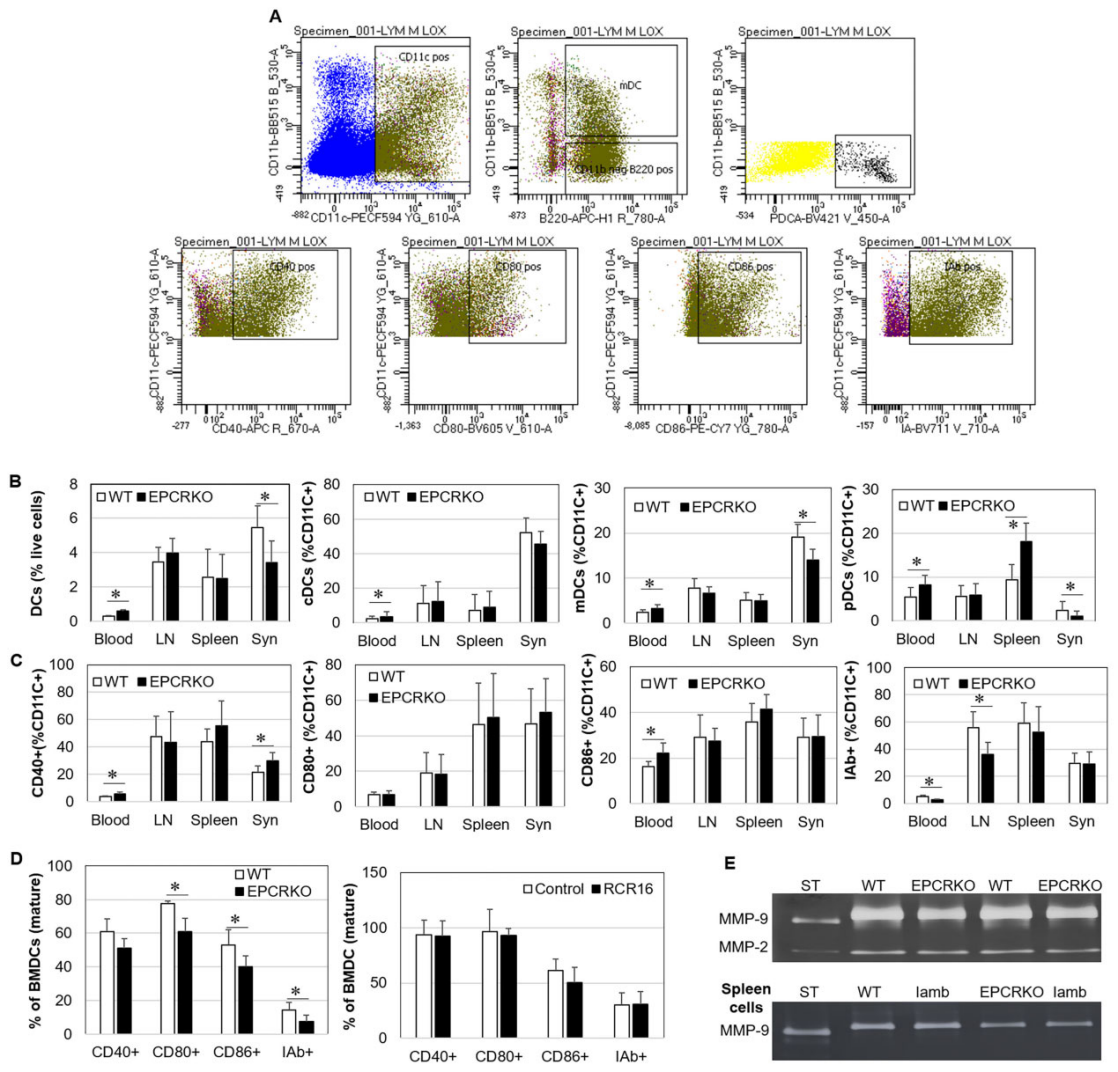
The *in vitro* and *in vivo* effects of EPCR on DCs. **(A)** The gating strategies for flow cytometry detection of DCs and DC subsets and the levels of CD40, CD80, CD86 and IAb on DCs within blood, lymph nodes (LN), spleen and synovium (Syn) from WT or EPCR KO mice with CIA. **(B)** DCs and their subsets cDCs, mDCs and pDCs within blood, LN, spleen and Syn from WT or EPCR KO mice with CIA and **(C)** levels of CD40, CD80, CD86 and IAb expression on DCs in these tissues (*n* = 5 mice). **(D)** Levels of CD40, CD80, CD86 and IAb on bone marrow (BM)-generated WT or EPCR KO mature BMDCs in response to RCR16 treatment for 24 h (*n* = 3). Data are shown as mean (s.d.). *P < 0.05, **P < 0.01. **(E)** MMP-2 and MMP-9 in the culture supernatants of BMDCs and spleen cells after 24 h incubation, detected by zymography. ST: MMP-2 and MMP-9 standard

DCs are professional antigen-presenting cells and play a critical role in the balance between tolerance and immunity [24]. DCs and their expression of CD40, CD80, CD86 and I-Ab MHC class II alloantigen (IAb) were detected by flow cytometry. Fig. 5A shows the gating strategies. DCs were higher in blood and lower in synovial tissues from EPCR KO mice with CIA when compared with WT mice (Fig. 5B). Among DCs, blood cDCs , mDCs and pDCs and spleen pDCs were higher and synovial mDCs and pDCs were lower in EPCR KO mice when compared with WT (Fig. 5A). In EPCR KO mice, blood DCs expressed higher levels of CD40 and CD86 and lower IAb, while lymph node DCs expressed lower levels of IAb and synovial DCs expressed higher levels of CD40 when compared with WT mice (Fig. 5C). *In vitro*, however, mature EPCR KO BMDCs expressed less CD80, CD86 and IAb when compared with WT and RCR16 treatment had no effect on their expression (Fig. 5D). Trafficking of DCs to lymph nodes is a crucial step in the pathogenesis of RA and DC-derived MMP-9 is the crucial factor for DC migration [25]. In this study, EPCR KO BMDCs produced lower levels of MMP-9 than WT cells. Similarly, EPCR KO spleen cells produced much less MMP-9 (Fig. 5E).

### Other immune cell populations in CIA

**Figure 6.**
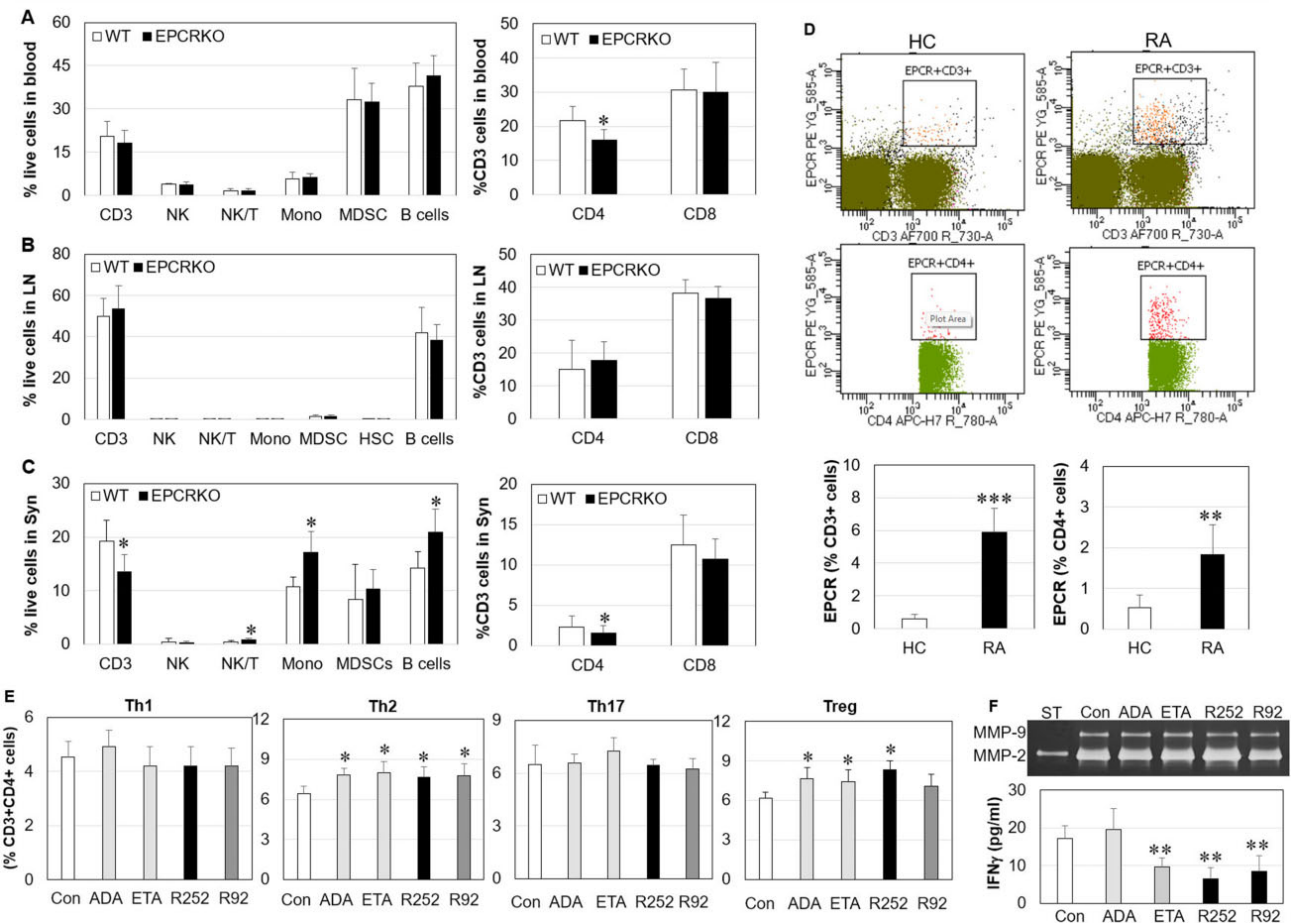
The effect of EPCR on immune cells from WT or EPCR KO mice with CIA and the effect of blocking EPCR on PBMCs from patients with RA. **(A-C)** The levels of immune cells including CD3, CD4 and CD8 T cells, NK cells, NK T cells (NK/T), monocytes (Mono), myeloid-derived suppressor cells (MDSCs) and B cells within blood, lymph node (LN), spleen and synovium (Syn) from WT or EPCR KO mice (*n* = 5 mice) with CIA, detected by flow cytometry. **(D)** EPCR expression on CD3 and CD4 T cells within PBMCs from patients with RA or matched HCs (*n* = 6). **(E)** The percentages of Th1, Th2, Th17 and Treg cells in CD3^+^CD4^+^ T cells within RA PBMCs treated with human EPCR blocking antibody RCR252 (R252), non-blocking antibody RCR92 (R92), TNF inhibitor ADA and ETA (all 10 µg/ml) for 24 h, detected by flow cytometry (*n* = 4). **(F)** MMP-2 and MMP-9 and IFN-γ in culture supernatants of RA PBMCs after 24 h treatment (*n* = 4). MMP-2 and MMP-9 were detected by zymography and IFN-γ by ELISA. Data are shown as mean (s.d.). ST: MMP-2 and MMP-9 standard; Con: control. *P < 0.05, **P < 0.01, ***P < 0.001 *vs* WT, HCs or Con

Other immune cell populations in blood, lymph node and synovial tissues from CIA mice were also examined by flow cytometry. CD3 T cells, NK cells, NK T cells, monocytes, myeloid-derived suppressor cells (MDSCs) and B cells within blood and lymph node did not show any significant differences in cell number between EPCR KO and WT mice with CIA, but EPCR KO mice had ∼20% fewer CD4 T cells in blood (Fig. 6A and B). Synovial tissue from EPCR KO mice contained fewer CD3 T cells and more NK T cells, monocytes and B cells; CD4 T cell numbers were ∼30% lower in EPCR KO synovial tissue when compared with WT (Fig. 6C).

### EPCR expression and its blocking effect on PBMCs from patients with RA

When detected by flow cytometry, CD3 and CD4 T cells within PBMCs from RA patients expressed ∼10 times [5.9 (s.d. 1.452) *vs* 0.6 (s.d. 0.284), *P* = 0.00087] and 3 times [1.63 (s.d. 0.74) *vs* 0.53 (s.d. 0.302), *P* = 0.0074] more EPCR, respectively, when compared with HC cells (Fig. 6D). Blocking EPCR and anti-TNF treatment stimulated Th2 and Treg cells but had no significant effect on Th1 and Th17 cells (Fig. 6E). Blocking EPCR also increased MMP-2 and inhibited IFN-γ production by RA PBMCs but did not affect MMP-9 (Fig. 6F). TNF inhibitors had no effect on either MMP-2 or MMP-9, but ETA, not ADA, inhibited IFN-γ (Fig. 6F). Similarly, the human EPCR non-blocking antibody RCR92 also displayed partial blocking effects as shown by the blocking antibody RCR252 on Th2 cells and IFN-γ. IL-4 and IL-17 were not detectable in the culture supernatants of RA PBMCs.

## Discussion

This study reveals that EPCR deficiency reduces the incidence as well as the severity of CIA, which is likely via modulation of immune cell activation, particularly T cells, and their secretion of cytokines and MMP-9, and EPCR is overexpressed by circulating T cells from RA patients. Blocking EPCR stimulated Treg cell and MMP-2 production but had an inhibitory effect on IFN-γ by RA PBMCs.

EPCR was originally identified as a specific receptor for PC/aPC [5] and mediates most of the cytoprotective function of aPC [5], with itself being anti-inflammatory. EPCR has been shown to inhibit Th17 cells, as mice with T cell–specific EPCR deficiency displayed more severe EAE [10]. Overexpression of EPCR protects transgenic mice from endotoxin-induced injury [26]. However, our data indicated that EPCR deficiency ameliorated murine CIA, consistent with our previous studies showing that EPCR is overexpressed by and can mediate RA synovial fibroblast invasion [8, 16]. Similarly, EPCR deficiency protects bacterial-induced lung injury [27], inhibits joint bleeding–induced inflammation [28] and deters the development of lupus and APS [7] in mice. Conversely, EPCR overexpression can predict poor outcomes of colorectal and lung cancers [12, 13] and poor treatment response of patients with lupus nephritis [15]. The underlying mechanisms of action of these conflicting functions of EPCR in different diseases are not clear but may be associated with its immune regulatory function.

In addition to acting as a receptor, EPCR is homologous to MHC class I/CD1 family proteins [9] and regulates T cell activation/differentiation [10, 29]. RA is a T cell–mediated autoimmune disease, where proportional and functional imbalances among T cell subsets play a critical role in RA pathogenesis [30]. Th1 and Th17 cells and their effector cytokines can initiate RA, while Th2 and Treg cells and their effector cytokines exhibit a protective role in both early and late RA [30]. The protective role of EPCR deficiency in CIA is likely achieved via regulation of these cells. In EPCR KO mice with CIA (Fig. 3), Th1 and Th17 cells were lower in lymph node and synovial tissues when compared with WT mice and, *in vitro*, EPCR deficiency decreased Th1 cells. Correspondingly, the Th1 cytokine IFN-γ was lower in circulation. IFN-γ can induce TNF and plays a dominant role in inflammatory RA [31, 32]. In contrast, EPCR deficiency increased Th2 cells and its signature cytokine IL-4. IL-4 inhibits Th17 differentiation, protects against cartilage and bone destruction and reduces disease activity in established CIA [33]. In addition, EPCR deficiency also reduced the circulating TGF-β1 and MMP-3 levels; both have been positively correlated with joint damage in RA [22, 34]. Consistent with a previous study showing that EPCR is a negative regulator of Th17 cells [10], we found that circulating Th17 cells were increased in EPCR KO mice with CIA (Fig. 3). However, higher levels of circulating Th17 cells did not result in more Th17 cells in lymph node and synovium.

Chemokines and chemokine receptors are responsible for the recruitment of inflammatory cells to lymphoid organs and sites of inflammation. Among them, CXCR3 and CXCR5 are important for the homing of Th1 cells into inflammatory synovium in RA [35, 36], while CCR6 is involved in the ingress of Th17 cells into rheumatoid joints [37]. CCR7 mediates the homing of T cells into lymph node and is associated with synovial lymphoid neogenesis [38]. The deficiency of CXCR5 on CD4^+^ cells results in complete abrogation of inflammatory arthritis in mice [39], while a lack of CCR7 on CD4^+^ T cells leads to reduced severity of EAE [40]. Lower levels of CXCR3, CXCR5, CCR6 and CCR7 on EPCR KO CD4^+^ T cells therefore likely contribute to the decreased number of CD4^+^ T cells and their inflammatory subsets in lymph node and synovium (Fig. 3D), leading to mitigated CIA in these mice.

The regulatory role of EPCR on DCs may also contribute to the mitigated arthritis in EPCR KO mice. EPCR-expressing DCs is a critical target of aPC therapy to reduce the mortality of endotoxemia in mice [41]. In RA patients, circulating DCs and their subsets are decreased compared with HCs [42] and decreased disease activity results in the re-establishment of the levels of DCs in the circulation in these patients [43]. RA synovial DCs are more mature than those residing in healthy synovial tissue [39]. Suppressing DC maturation and migration attenuates inflammatory arthritis in mice [44, 45]. EPCR deficiency resulted in increased DCs in circulation and decreased DCs in synovial tissue. *In vitro*, EPCR KO BMDCs were less mature and produced less MMP-9 compared with WT cells (Fig. 5). MMP-9 is the key molecule for DC migration and blocking MMP-9 suppresses the development of CIA by inhibition of DC trafficking [25].

The binding of PC to EPCR promotes the activation of PC to aPC [46]. In this study, under unchallenged conditions, EPCR deficiency did not affect aPC activity. In CIA, however, EPCR KO mice generated less endogenous aPC within the synovium when compared with WT mice (Fig. 2). This is probably associated with less inflammation in the joints of EPCR KO mice compared with WT. In the RA joint, increased inflammation promotes the production of other EPCR ligands, such as sPLA2, T cell receptor [6] and aPL antibodies [7]. These ligands compete with PC/aPC to bind to EPCR, thus rendering EPCR unable to regulate aPC levels at inflammatory sites. Instead, other PC activators such as thrombin [47] may contribute to higher levels of aPC in RA synovium, as the thrombin–thrombomodulin complex can activate PC to aPC even in the absence of EPCR [48]. This is in agreement with a previous study that showed aPC activity/expression in the joints from patients with RA was markedly higher when compared with OA [49].

Additionally, similar to psoriasis [11] and RA synovial fibroblasts [8], higher levels of EPCR were also found on RA circulating T cells, and blocking this receptor can stimulate Th2 and Treg cells and anti-inflammatory MMP-2 production [50] in RA PBMCs. These data suggest that this receptor may be inflammatory and is a potential drug target for RA.

In summary, this study demonstrated that deficiency of EPCR prevented inflammatory arthritis in mice. This is likely achieved by modulating the activation and migration of T cells and DCs. Targeting EPCR or its associated downstream signalling pathways may constitute a novel strategy of future therapy for RA and other similar autoimmune diseases.

## Data Availability

The data underlying this article will be shared upon reasonable request to the corresponding author.
